# External quality control in laboratory medicine. Progresses and future

**DOI:** 10.1515/almed-2022-0058

**Published:** 2022-08-17

**Authors:** Carmen Ricós, Pilar Fernández-Calle, Carmen Perich, Sverre Sandberg

**Affiliations:** Sociedad Española de Medicina del Laboratorio (SEQC^ML^), Barcelona, Spain; Departamento de Medicina del Laboratorio, Hospital Universitario La Paz, Madrid, Spain; Norwegian Organization for Quality Improvement of Laboratory Examinations (NOKLUS), Hospital Universitario Haukeland, Bergen, Norway

**Keywords:** external control, performance specifications, quality assurance

## Abstract

**Objectives:**

An external quality control program distributes same control samples to various laboratories and evaluates results obtained with a common criterion. The aim of this work is to summarize the evolution of various types of external programs, to point out the progresses ant to preclude practical consequences of the participant laboratories.

**Content:**

The material consists on a brief revision of the different types of external programs that have been used for the last forty years. The method is the critical analysis of the strong and weak points of each program model, from the light of our experience. External quality assessment (EQA) programs were initiated at half the XX century, evidencing big discrepancies among laboratory results. EQA were developed in various countries and some mechanisms to harmonize them were proposed: to establish common performance specifications derived from biological variation, to use EQS as educational tool. Since the 2000 important advances were seen: to focus EQA to assure the adequate clinical use of laboratory tests, to use commutable controls, to harmonize the different EQA models, to promote a forum for co-operation and exchange of knowledge on quality-related matters for EQA organizers.

**Summary and Outlook:**

To participate in an EQA with commutable-reference method assigned values controls allows to know the real inaccuracy of results and their impact on patient’ samples. To participate in a EQA with non commutable controls allows to know whether the individual laboratory performance agrees with that from other laboratories using same analytical method.

## Introduction

Laboratory medicine is a medical science pioneer in development and application of external quality control of analytical procedures. An external quality control program consists in a systematic distribution of several control samples among a number of laboratories and an evaluation of results obtained by an organization external to the participant laboratory. The generally accepted term for this activity is external quality assessment (EQA) [1].

Another term used to refer to these activities is proficiency testing that is something practically identical to external quality control that formally recognizes the attainment of good results by the participant [2, 3].

This study is focused only on the external quality control of the analytical phase of the medical laboratory.

The aim of this study is to summarize the evolution of external programs, exemplified with the Spanish EQA evolution, to signal the progresses and to deduct practical consequences for the participant laboratory and for the external program organizers.

## Materials and methods

The material for this study is a brief revision of the different types external programs that have been used for the last forty years.

The method is the critical analysis of the strong and weak points of each program model, from the light of our experience.

## Results

### Preliminary history

The first program was created by Bellk and Sunderman in the USA in 1947 [4]; aqueous solutions of 9 biochemistry analytes were distributed to around 60 labs, and evidenced that, on the average, 15% of results were identified as gross errors, around 50% were unsatisfactory and 35% were considered to be satisfactory.

First program in hematology was created in 1969 by Lewis and Burgess [5] and the emerge of this practice arrived in the 70 s in the Atlanta Conference [6] and immediately after the IFCC published a provisional recommendation on external quality assessment (EQA), also known as inter-comparison schedule, that became an approved recommendation in 1983 [7]. The main objectives were:

– To estimate the inaccuracy of results obtained by every participant laboratory.
– To verify the imprecision for schemes where the control material was analysed several times, comparing to that obtained by the other participants).
– To evaluate acceptability of results.

Briefly, external control of extra-analytical phases was created by the College of American Pathologists (CAP) [8, 9] and the Spanish Society of Laboratory Medicine (SEQC) offers several programs since 2008 [10], [11], [12].

SEQC biochemistry programs were initiated in 1977, by means of collaboration with the *Société Française de Biologie Clinique*, with 60 Spanish laboratories involved.

The first SEQC EQA organized by its own was developed in 1980; it was focused on serum biochemistry and was evaluated and accredited by the World Health Organization in 1984. A multi-disciplinary program (biochemistry, hematology and microbiology) was developed between 1989 and 2002 by an agreement among the Spanish Association of Hematology and Hemotherapy and with a group of microbiologists. Later on various programs were developed, such as urine biochemistry, hormones, proteins, gases, glycohemoglobin, cardiac markers, tumor markers, therapeutic drugs, and abuse drugs.

The SEQC External Programs Committee was integrated in international working groups since the beginning, with the aim to continuously improving their programs and to maintain them in the first line knowledge’s, within the resources available in our setting.

Up to that moment the analysis of results from participants was merely descriptive, by means of a statistical study of the dispersion of results and the comparison of individual results with a global consensus mean, and each EQA organizer had established its own criteria for analysis and for compliance.

In the 90 s the *Standards Measurement and Testing Programme* of the European Communities developed some criteria to standardize the existing EQAs in that moment [13]. These criteria were:

– To define quality specifications for EQA.
– To promote use of homogeneous analytical systems (calibrator, reagents, and instrument from same manufacturer).
– To select the best control material possible.
– To evaluate individual laboratory performance and participant methods performance using similar criteria among all EQA.
– To focus on educational EQA.

The use of analytical quality specifications based on biological variation both for internal quality control [14] and for external quality assurance was recommended; this last based in the evidence of wide discrepancies among European EQA programs, i.e., cholesterol, where deviations to the target value ranging from 3 to 18% were accepted, depending on the EQA organizer [15].

The requirements of control materials for serum programs were also explained, concluding that the appropriateness of materials in respect of scheme design and objectives is important [16].

To evaluate the accuracy of participating laboratories, establishing target values of control materials by the use of recognized reference method was promoted [17] and a network of such type of laboratories was then created [18].

All this knowledge was internationally recognized in an ISO standard: ISO-Guide 43 Proficiency Testing by interlaboratory comparisons [19].

In 1998 the European Directive 98/79/EC [20] established that EQA should verify the harmonization among laboratory tests results using the analytical systems available in the market. According to this standard, an EQA program made interlaboratory comparisons designed and performed to assure some of the following aspects: the evaluation of the participant laboratory performance, the evaluation of the analytical methods, to establish an *in vitro* analytical systems vigilance, as well as continuous education training and help for the participants.

In 2002 the IFCC provided a series of recommendations to EQA organizers, so their technical competence in their vigilance role could be accredited [21].

At that moment a new concept of EQA was defined by the IFCC, *External Quality Assurance Program* (EQAP), to differentiate from that up to then have been called external quality assessment scheme (EQAS). The word “assurance” in this context is different than “assessment” because it is not simply an evaluation of the analytical performance but includes the interpretation of test results and gives advice to clinicians about the diagnostic capacity of them. Moreover, another objective was added: training and continuous education of laboratory professionals. The aim of EQAP is pushing quality of the laboratory service for patient benefit.

The abbreviation EQAP is however more seldom used nowadays and most EQA providers will claim that they also do “assurance”.

EQALM, the European Organization for External Quality Assurance Providers in Laboratory Medicine, uses the word “assurance” in its name underlining that this is actually what is done by the EQA providers. EQALM is an umbrella organization for European EQA organizers in laboratory medicine. EQALM provides a forum for co-operation and exchange of knowledge on quality-related matters especially with regard to EQA programs in Europe.

The original term EQA in this revision is used in generic sense, because it is not our aim to point out the differences among the various recognized abbreviations of external quality control in laboratory medicine.

### EQA advances in the XXI century

During fist two decades of the XXI century various aspects concerning EQA improvement were developed:

(1) Nature of control material: commutability and values assignment.
(2) Differentiation of types if EQA according to its evaluation capacity.
(3) Establishment of Analytical Performance specifications for evaluating participants.
(4) Harmonization among EQA.
(5) Vigilance of the quality of the current available analytical methods.

Each of these aspects is commented here.

#### Nature of control material: commutability and values assignment

Commutable controls have same matrix as for patient samples and, consequently, its behavior in front the analytical routine methods is the same for testing the same analyte.

As an example, the Dutch EQA organization (SKML) developed in the 2000 a program for 6 enzymes using commutable control that was distributed to participants together with the regular program (non-commutable control). Inter-laboratory imprecision was lower in the first case (CV between 2.2 and 4.9% for the different enzymes) than in the second case (CV between 4.6 and 10.8%) [22].

The SEQC-EQA organization established an agreement with SKML to distribute their commutable control of the basic biochemistry program, once per year since 2015 up to now.

Ceriotti demonstrated in 2014 that if commutable-reference values control samples are used, the uncertainty of test results can be verified and adequate corrective actions can be taken, accordingly [23].

The way for an EQA to surely verify the accuracy of participant results is to distribute human samples simply frozen (without any other manipulation), with verified commutability and with values assigned by the reference laboratories listed in the *Joint Committee for Traceability in Laboratory Medicine* (JCTLM) (https://www.jctlm.org/), which use reference methods when existing [24].

Assigning values with reference methods to non-commutable control sample implies an inadequate use of resources, because routine methods may have different behavior with these materials than with patient samples and, consequently, the information produced may not be translated to patient results.

#### Types if EQA according to its evaluation capacity

The European standard EN 14136 published in 2004 does not impose a single organization model for EQA in Europe, but it insists that EQA should provide useful information to monitor analytical performance of routine methods as, for example, to unequivocally identify individual analytical procedures and, mainly, to distinguish between the performance characteristics of an individual procedure and those attributable to its users [25].

The *Clinical and Laboratory Standards Institute* (CLSI) from USA in 2008 outlined the EQA characteristics and insisted on its educational role as, for example, by informing the participant laboratory about the possible repercussion of its incorrect result on patients [26].

Miller and cols. Identified in 2011 the key factors to evaluate EQA strengths: to know the commutability of their control materials and the procedure used to assigns their target values: Six categories were established, according to the valuation capacity of EQA [27]:

– Categories 1 and 2: use commutable control materials with assigned values by certified reference methods. The first category tests replicates of control samples and, consequently, verifies accuracy and reproducibility of the participant laboratory, the peer group (same method, instrument and reagent users) and the method group, as well as, verifies traceability to higher order standards. It assures standardization among laboratories. The second category verifies laboratory accuracy but no reproducibility.
– Categories 3 and 4: use commutable control materials but without values assigned by certified reference methods. The first one uses replicated controls, thus evaluating reproducibility of the individual laboratory and of the peer group. The second one uses single control results and, consequently, only evaluates reproducibility between groups. Both categories compare deviation between peer groups, but are not able to evaluate neither accuracy nor traceability to higher order standards.
– Categories 5 and 6: use non-commutable and non-reference values control materials. Category 5 uses replicated controls thus verifying laboratory and group reproducibility; category 6 only verifies laboratory reproducibility.

Various EQA providers have programs where they claim to have commutable samples; however, a number of EQA organizations use categories 5 and 6. Moreover, no reference materials neither reference methods exist for a number of analytes commonly tested in laboratory medicine.

#### Performance specifications for evaluating participants

As it has been mentioned previously, in 1996 the BCR European group had seen that a 56% of EQA programs used state of the art, 25% expert’s opinions (diverse and divergent) and 19% biological variation [15]. Twenty years after, in 2017, Jones et al. saw through an enquiry 42% use biological variation and 38% the state of the art [28].

The Australian program for hormones used in 2004 specifications derived from biological variation to evaluate imprecision, using fixed limits in EQA (same limit for each analyte, independently of the methodology used facilitates identification of the source of error in the laboratory, clarifies evaluation of analytical methods and unifies information obtained by different EQA. This work points up the use of non-commutable controls as a limitation, because information produced may be non-transferable to human specimens [29].

From a meeting of Europe, Israel and South Africa experts held in 2011 [30], where the specifications to be used in EQA were deeply debated, it was concluded that those derived from state of the art are useful for non-analytical performance, whereas those derived from biological variation are recommended for analytical performance; moreover, laboratories should push *in vitro diagnostic* providers so their systems reached such specifications.

In 2015 Jones alerted the lack of harmonization among specifications used in EQA even 15 years after the international consensus of Stockholm. He insisted that EQA should inform their participants on the specifications used and the reasons why they had been chosen [31].

In the 1st EFLM Strategic Conference (Milan 2014) one goal for EQA was agreed: to harmonize specifications for evaluating participants; in this way, same type of messages about the quality of laboratory service would be expanded, independently of the laboratory performer and the EQA selected.

The *EFLM Task Finish Group – Analytical Performance Specifications for EQAS* recommended that all EQA should inform their participants about 6 aspects [32]:

– Matrix and commutability of control materials distributed.
– Method used to assign values to control materials.
– Data set for application of APS (individual laboratory deviation, peer group deviation, etc.).
– Analytical variable evaluated (imprecision, bias, total error).
– Type of specification (biological variation, state of the art, etc.).
– Reason for having chosen the specification.

There are some EQA for tests expressed in nominal and ordinal, in which labs are evaluated in the basis of the percentage of correct answers obtained during a cycle. Whether such ordinal scale tests are converted into quantitative tests, specifications are the same as have been described in previous paragraphs [33].

#### Standardization among EQA

The five pillars for standardization had been identified [34], [35], [36] as:

– Certified reference materials.
– Reference measurement procedures.
– Recognized reference laboratories.
– Reference interval agreed.
– Comparable EQA programs.

About the 5th pillar, that concerns EQA, the authors insisted one decade ago that, to reach standardization among medical laboratory measurements, EQA should be comparable.

Consequently, the capacity of a category 1 EQA to evaluate standardization of laboratory performances was investigated by Jansen et al. in a study with laboratories from The Netherlands, Portugal, Spain, and United Kingdom. Total analytical error was evaluated based on biological variation criterion and important discrepancies were seen for 11 out of the 18 analytes studied (calcium, chloride, magnesium, sodium, ALT, amylase, AST, LDH, HDL cholesterol, creatinine, and total proteins). For many of them, discrepancies were related to the instrument used and for some even among users of same instrument, [37, 38].

Also, the studies by De Grande et al. (Empower project) [39] making comparisons with panels of frozen single-donation samples, as well as monitoring of patient percentiles sent by a number of laboratories give a realistic view on assay comparability.

Results obtained during 4 years of a category 1-EQA experience distributed in Spain were evaluated by Ricós et al. [28]. Only 2 out of the 18 analytes studied could be considered standardized (CK, potassium) because bias among peer groups reached the corresponding specification derived from biological variation.

There was no standardization for 8 analytes, due to diverse reasons:

– ALP and Proteins with all labs sing same method showed differences among instruments.
– ALT, AST, amylase, creatinine, GGT, and LDH evidenced high deviations for some concrete methods: ALT and AST without pyridoxal phosphate, amylase with malto-triose substrate, creatinine with Jaffé method, GGT with<4 mmol substrate, and LDH with reverse method (pyruvate-lactate).

In 2021 (non-published data) the 8 non-standardized analytes without apparent reason found in previous years (2015–2019) were studied, focusing only in concentrations of clinical interest:

– Magnesium and urate were seen to be standardized at these concentrations. Same method was used for urate in all labs (uricase-POD) and three methods for magnesium (xylidyl blue, methyl thymol blue, and enzymatic), indicating that the variety of methods is not a limiting factor for standardization. Both analytes were traceable to reference methods (IDMS in urate, atomic absorption in magnesium), showing as the traceability to a method (that obviates the possible matrix effect of a reference material), favors standardization.
– Bilirubin, calcium and glucose were well standardized only in some years. The discontinuity of standardization could be due to changes of routine calibrator lots or of some intermediate materials in the manufacturer’s reference system as had been seen by Braga et al. [40], or due to a too permissive uncertainty admitted by the manufacturer’ intermediate materials [41].
– For chloride and sodium both measured by ISE, standardization is attained sometimes or never, respectively, without recognized reasons.

#### Vigilance of the routine analytical methods used

The vigilance role given to EQA by the European directive 98/79/EC was early applied by the Belgian program during 10 years, evidencing the following “surprises” [42]:

– Inadequate discriminant values and biological reference values described in some manufacturers’ inserts.
– Unspecific reactions in some reagent lots.
– Corrosion producing interferences in some analyzer’ dispensers
– Reagent kits with inadequate performance.

**Figure 1:**
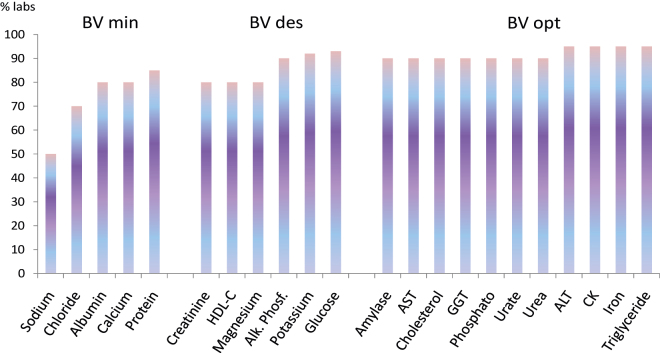
Percentage of laboratories reaching specifications for total analytical error based on biological variation in SEQC biochemistry program. Data obtained from Perich et al. [43].

**Figure 2:**
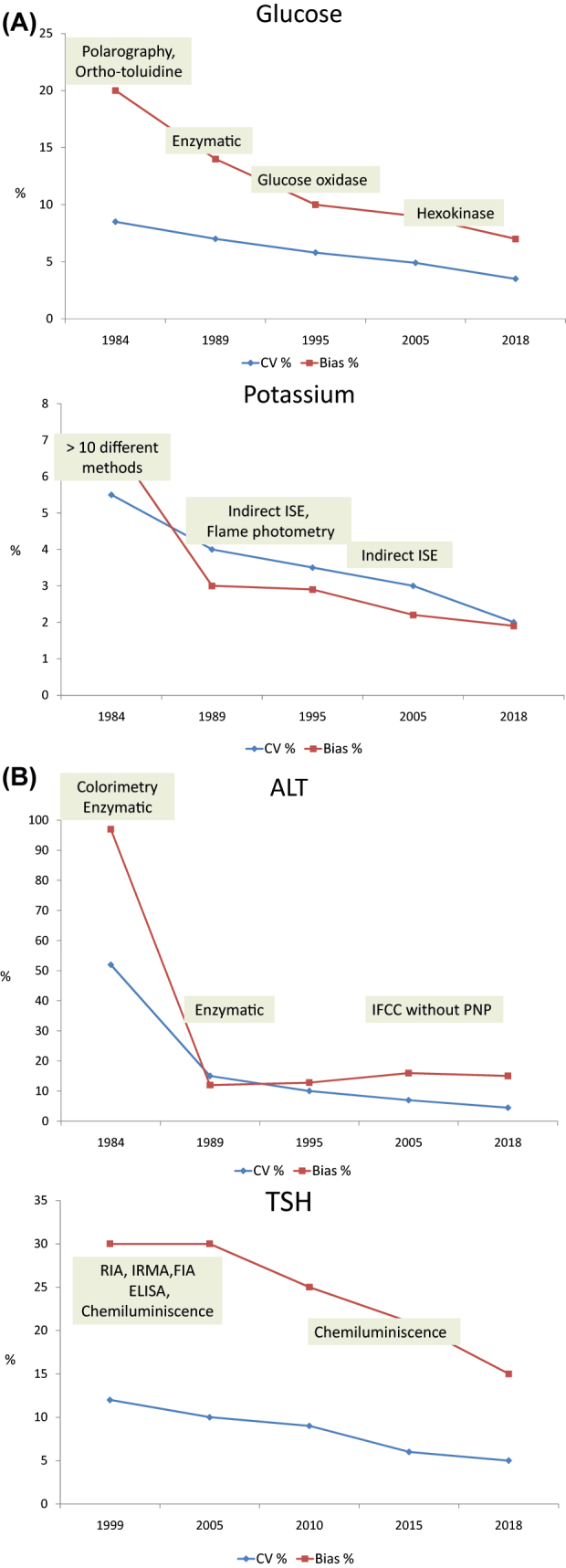
Coefficient of variation (%) and bias (%) evolution. (A) Glucose and potassium (B) ALT and TSH. Data obtained from Perich et al. [43].

Evolution of results obtained in the SEQC EQA programs using lyophilized control sera was studied by Perich et al. [43] for more than 30 years (1981–2018). An improvement was seen, because individual laboratory deviations to target values decreased with time; also in 2018, the program of biochemistry components showed that the 90% of participants reached specifications for total error derived from biological variation and even more than 50% did the same or analytes with strong physiological regulation (Figure 1). Also, reduction of inter-laboratory imprecision and bias was observed, mainly due to changes of the analytical methods used by participants (Figure 2A and B). As a negative note, persistency of obsolete of poorly specific methods was detected, when better methods are available, i.e. creatinine with Jaffé or transaminases with IFCC based method but without pyridoxal-phosphate).

Inter-laboratory imprecision was the same order than of the Dutch EQA (SKML) [44], the Belgian Empower Project [45] by the German program (German Referenzinstitut für Bioanalytik) [46]. However, some difficulties to easily compare various EQA were found; these are:

– Data was only available for programs publishing results in index journals or in websites available for non-participants.
– Discrepant criteria used to create peer groups.
– Different calculation made on the data obtained.
– Diverse specifications used to evaluate participants.

### Strong points and weak points of EQA

Three questions concerning EQA were posed by Ceriottiy and Cobbaert in 2018 [47]:

– Are external quality assessment schemes (EQAS) really fit for purpose?
– Are all schemes equivalent and sufficiently harmonized?
– Is the role of EQAS similar and necessary in all branches of laboratory medicine?

The answer to first question is no, because many EQA do not verify laboratory trueness because non-commutable and reference-value assigned controls are used; these EQA only assess comparability among peer groups.

The answer to second question is, again, no: different types of control materials and diverse criteria to evaluate performance are used; this may be due to the various types of EQA existing, from those that are mandatory with punishment for non-compliant laboratories to educational EQA that push to improve laboratory performance. This situation could improve if accreditation of EQA ISO 17043:2010 [48] standard was implemented.

The answer to third question is yes: it is clearly necessary to inform about trueness and reproducibility of all medical laboratory tests.

The key aspects that all EQA should comply to facilitate laboratories to be accredited by the ISO 15189 standard were studied by Sciacovelli et al. [49]:

– To define the best performance specification for each analyte and these were adopted by all EQA.
– To help laboratories to find alternatives for comparison when no EQA were
– To clearly describe in their reports the reasons for unsatisfactory performance, mainly if they could be due to the own laboratory or to the analytical procedure used.

Badrick and Stavelin [50] noted that because the coexistence of two general types of EQA (mandatory and educational), a laboratory participating in both types can reach acceptable performance in one of them but inacceptable in the other, for same measurand. These authors proposed to create an entity for leading harmonization among EQA and propose EQA the already existing *European Organization for External Quality Assurance Providers in Laboratory Medicine* (EQALM) (http://www.eqalm.org), which has the confidence of many EQA organizers.

The ability to aggregate data from four countries EQA (Norway, The Nederland, United Kingdom and USA) was investigated by Van der Hagen et al. [51] for creatinine, using commutable-reference method value controls. The feasibility for aggregation was verified if of methods and analytical systems was the same in all EQA. A common description of commutability of controls distributed, as well as some information from participants such as calibration and reagent lots should be a requirement to optimize comparison among the analytical systems used by medical laboratories. Recently, the International Consortium for Harmonization in Laboratory Medicine (ICHCLR) and The European Organization for External Quality Assurance Providers in Laboratory Medicine (EQALM), have joined forces for an initiative called HALMA to assess harmonization of measurands through aggregated EQA data on an international basis (http://www.eqalm.org/site/halma/halma.php)

Pros and cons of the most used EQA programs have been manifested by Jones et al. [52] and are summarized in Table 1, which was presented by González-Tarancón et al. in a SEQC – Academia SEQC of April 2022 [53].

**Table 1:** Pros and cons of the most used EQA programs.

Type of EQA	Pros	Cons
1 and 2	– To know real imprecision and bias, and to extrapolate to patients’ results.	– Expensive
	– To verify standardization of methods and interchangeability of results	– Maintenance requirements
	– To identify deficient laboratories and methods	
	– To eliminate non standardized methods	
	– To verify validity of patients’ results for being further used n indirect studies (big data).	
5 and 6	– To compare between equals	– Do not know accuracy
	– To verify harmonization	– Do not allow standardization
	– Cheaper	
	– To monitor individual laboratory	
	– To monitor manufacturer	

Obtained from Gonzalez-Tarancón et al. [53].

### Recommendations to EQA participants

The CLSI (CLSI-GP 27-A2) provides some recommendations to adequately use EQA programs, such as [26]:

– To revise each EQA report for observing the laboratory deviation from the peer group and to draw figures for monitoring the various deviations in a cycle, for each analyte.
– To see at peer groups dispersion and be aware of the most precise group.
– To study the type of errors made and investigate whether they are clerical, due to the analytical procedure implemented, to the method used, to personnel abilities or unexplained.
– To implement corrective actions and to evaluate their effect when seeing the following EQA report.

Other things can be made by a laboratory participating in a cathegory 1 program:

– To surely know the effect or the impact that every deviation has on their patients (calibration, lot to lot variation, etc.)
– To assess its inaccuracy by analyzing its deviation against the reference value.
– To be aware of the most accurate method available.

When no EQA is available for determined analyte, it is possible to change patient samples with other labs and to interpret results following the CLSI-EP-31-A-IR [54] recommendations.

### How the laboratory should select an EQA

Miller and Sandberg [33] suggest the following six questions to laboratories:

– How similar the control material is compared with patient samples?
– Is it a commutable control?
– How many control replicates are measured in each EQA event?
– How the target value of control has been established?
– How many participants are in the peer group?
– How performance specifications have been setting up?

If possible, laboratory should select an EQA with commutable-reference method values; these methods exist for approximately 110 analytes [55]. In this way, the lab knows the accuracy of its performance and the EQA verifies what methods are standardized. It is of vital interest for anaytes that are interpreted against a clinical decision value.

When participating in an EQA with non-commutable controls the laboratory is compared with its peer group, allowing sharing population-based reference values. However, within the peer group discrepancies in control results due to different reagent lots may be seen, even though patient’ results could give similar results.

If the EQA design includes testing of replicated control samples, reproducibility of laboratory test results will be informed.

Target values uncertainty of non-commutable controls is higher than that of commutable ones, because the imprecision of routine methods is higher than of reference methods and it depends on the number of labs integrating the peer group.

### EQA in point-of-care testing (POCT)

POCT tests should be managed with same quality assurance strategies that the central laboratory (internal quality assessment and EQA), though clinical decision can be taken on the basis of POCT results and this information has to be managed by the central laboratory.

However, in some cases, POCT performance is different than that of the central laboratory (i.e., HbA1c) and, moreover, POCT tests may not be directed to the same clinical use; so, these analytical systems might constitute a particular group with their own performance specifications targets.

Ideally, EQA specifically designed for POCT networks should be created. NOKLUS has a lot of experience on this area, with high commitment and great outcomes reached in the information provided and their educational programs. Other organizations such as INSTAND (Germany), WEQAS for cooximetry and bilirubin (Wales) or Labquality for capilar glucose (Finland) offer also this type of specific programs.

### What should the laboratory do?

When the lab is responsible of other satellite labs with analytical procedures adjusted among them with correction factors, the results sent to EQA organization must be previously with the factors cancelled; otherwise, the peer group to be compared would be inexistent.

In any case the laboratory should adjust their results on the basis of the EQA reports, because the traceability to higher order standards given by the manufacturer of the analytical systems is lost.

## Conclusions

Laboratories should be conscious that:

– To know real accuracy of tests results and patient’ impact, as well as calibrators traceability, participation in EQA with commutable control material where the target value preferentially is set by a reference measurement procedure.
– To compare its performance with that of other laboratories using same measuring systems, participation in EQA with non-commutable controls is enough; however, they should be commutable between reagent lots.

EQA organizers should consolidate:

– To offer programs with commutable–reference method assigned controls, which verify standardization among laboratories.
– To inform that if non-commutable controls are distributed, they only evaluate harmonization within the same group of measuring systems.
– To encourage harmonization among EQAS.
– To aggregate their results with those from other EQA, to promote harmonization at a wide scale.
– To ask participants recording the reagent and calibrator lots, in addition to analytical system used for each analyte; the reason is that routine methods can give different results with different lots are used.
